# Mild Coronavirus Disease 2019 (COVID-19) Is Marked by Systemic Oxidative Stress: A Pilot Study

**DOI:** 10.3390/antiox10122022

**Published:** 2021-12-20

**Authors:** Larissa E. van Eijk, Adriana Tami, Jan-Luuk Hillebrands, Wilfred F. A. den Dunnen, Martin H. de Borst, Peter H. J. van der Voort, Marian L. C. Bulthuis, Alida C. M. Veloo, Karin I. Wold, María F. Vincenti González, Bernardina T. F. van der Gun, Harry van Goor, Arno R. Bourgonje

**Affiliations:** 1 Department of Pathology and Medical Biology, University Medical Center Groningen, University of Groningen, 9713 GZ Groningen, The Netherlands; l.e.van.eijk@umcg.nl (L.E.v.E.); j.l.hillebrands@umcg.nl (J.-L.H.); w.f.a.den.dunnen@umcg.nl (W.F.A.d.D.); m.bulthuis01@umcg.nl (M.L.C.B.); h.van.goor@umcg.nl (H.v.G.); 2 Department of Medical Microbiology and Infection Prevention, University Medical Center Groningen, University of Groningen, 9713 GZ Groningen, The Netherlands; a.tami@umcg.nl (A.T.); a.c.m.veloo@umcg.nl (A.C.M.V.); k.i.wold@umcg.nl (K.I.W.); m.f.vincenti.gonzalez@umcg.nl (M.F.V.G.); b.t.f.van.der.gun01@umcg.nl (B.T.F.v.d.G.); 3 Department of Internal Medicine, University Medical Center Groningen, University of Groningen, 9713 GZ Groningen, The Netherlands; m.h.de.borst@umcg.nl; 4 Department of Critical Care Medicine, University Medical Center Groningen, University of Groningen, 9713 GZ Groningen, The Netherlands; p.h.j.van.der.voort@umcg.nl; 5 Department of Gastroenterology and Hepatology, University Medical Center Groningen, University of Groningen, 9713 GZ Groningen, The Netherlands

**Keywords:** oxidative stress, free thiols, COVID-19, redox

## Abstract

Oxidative stress has been implicated to play a critical role in the pathophysiology of coronavirus disease 2019 (COVID-19) and may therefore be considered as a relevant therapeutic target. Serum free thiols (R-SH, sulfhydryl groups) comprise a robust marker of systemic oxidative stress, since they are readily oxidized by reactive oxygen species (ROS). In this study, serum free thiol concentrations were measured in hospitalized and non-hospitalized patients with COVID-19 and healthy controls and their associations with relevant clinical parameters were examined. Serum free thiol concentrations were measured colorimetrically (Ellman’s method) in 29 non-hospitalized COVID-19 subjects and 30 age-, sex-, and body-mass index (BMI)-matched healthy controls and analyzed for associations with clinical and biochemical disease parameters. Additional free thiol measurements were performed on seven serum samples from COVID-19 subjects who required hospitalization to examine their correlation with disease severity. Non-hospitalized subjects with COVID-19 had significantly lower concentrations of serum free thiols compared to healthy controls (*p* = 0.014), indicating oxidative stress. Serum free thiols were positively associated with albumin (St. β = 0.710, *p <* 0.001) and inversely associated with CRP (St. β = −0.434, *p* = 0.027), and showed significant discriminative ability to differentiate subjects with COVID-19 from healthy controls (AUC = 0.69, *p* = 0.011), which was slightly higher than the discriminative performance of CRP concentrations regarding COVID-19 diagnosis (AUC = 0.66, *p* = 0.042). This study concludes that systemic oxidative stress is increased in patients with COVID-19 compared with healthy controls. This opens an avenue of treatment options since free thiols are amenable to therapeutic modulation.

## 1. Introduction

Coronavirus disease 2019 (COVID-19), caused by severe acute respiratory syndrome coronavirus 2 (SARS-CoV-2), is an ongoing pandemic that continues its morbidity and mortality rates to mount globally. Although primarily causing mild respiratory complaints in the majority of individuals, some patients may develop severe pulmonary and systemic disease with clinical scenarios of acute respiratory distress syndrome (ARDS) and multi-organ failure (MOF) [1]. Despite the worldwide scientific endeavor to comprehend this serious viral respiratory disease, its complex pathogenesis remains incompletely understood. Oxidative stress has been implicated to play a pivotal role in COVID-19 pathophysiology and is associated with other underlying mechanisms involved, including hyperinflammation, coagulopathy, and hypoxia [2,3].

Severe COVID-19 is marked by extensive inflammatory pathology that can affect various organs within the body. Immune cell infiltration leads to the reduction of antioxidants and to the production of reactive oxygen species (ROS) that are thought to induce an inflammation-driven oxidative storm in COVID-19, thereby altering the redox balance. Oxidative stress itself may exacerbate inflammation through activation of transcription factors and pro-inflammatory genes, creating a vicious cycle of self-perpetuating oxidative stress and hyperinflammation [4]. Another major player in the pathophysiology of COVID-19 is tissue hypoxia that may be induced by thrombosis or respiratory distress-related hypoxemia, which in turn also interrelates with oxidative stress. Taken together, a systemic disbalance in redox status is considered to be part of the complex pathogenesis of COVID-19, providing a therapeutic target that could especially benefit individuals with an already impaired redox balance [5,6]. Although strong on theoretical grounds with upcoming studies providing direct evidence of oxidative stress in COVID-19, the exact role of oxidative stress in COVID-19 remains unclear and biomarkers to evaluate the redox status in COVID-19 are urgently needed [7,8].

Serum free thiols (R-SH, sulfhydryl groups) have previously been shown to comprise a direct and reliable reflection of the systemic redox system [9,10,11]. Free thiols constitute the main biological targets of ROS both inside and outside of cells, govern a variety of protein functions with downstream effects on short-term and longer-term biological adaptations, and they possess potent antioxidant buffering capacity [12,13]. Free thiols can be oxidized by locally produced ROS and are typically decreased in conditions associated with oxidative stress [14]. Furthermore, free thiols have been demonstrated to correlate with disease severity and to predict clinical outcome in several conditions [9,15]. Assessment of extracellular free thiols is an easy, robust, minimally invasive and reliable tool for quantification of the extent of systemic oxidative stress. In light of these considerations, we hypothesized that COVID-19 will be associated with reduced concentrations of free thiols, reflecting ongoing systemic oxidative stress in this disease. In fact, other studies have recently demonstrated lower free thiol concentrations in COVID-19 as compared to healthy controls [7,8], by using a spectrophotometric method developed by Erel and Neselioglu to measure native thiol, total thiol, and disulphide levels [16]. We hypothesized that this systemic redox imbalance is present even in mild COVID-19 not requiring hospital admission. As disease progression is marked by hyperinflammation and hypoxia—both associates of oxidative stress—we additionally hypothesized that free thiol concentrations may correlate with disease severity.

In this study, we aimed to determine serum free thiol concentrations particularly in patients with mild COVID-19 and age-, sex-, and body-mass index (BMI)-matched healthy controls while studying their associations with relevant clinical and biochemical parameters.

## 2. Materials and Methods

### 2.1. Study Population

This study used data from the COVID-HOME study, which is a prospective population-based cohort study carried out in the Northern part of the Netherlands primarily aimed at gaining insight into COVID-19 in subjects who are not hospitalized [17]. For the current study we included 36 participants (both non-hospitalized and hospitalized) who all had an established COVID-19 diagnosis based on a positive PCR test. Seven (19.4%) participants eventually required hospitalization. None of the subjects were vaccinated against SARS-CoV-2. The study was approved by the Institutional Review Board (IRB) of the University Medical Center Groningen (UMCG) (IRB no. 2020/158). All subjects provided written informed consent for use of their data and biomaterials. Data and samples were shared in a pseudonymized manner according to the exchange of Personal Data as defined in Regulation (EU) 2016/679 of the European Parliament and of the Council of 27 April 2016 (GDPR). Serum samples from 30 age-, gender- and BMI-matched healthy control patients without COVID-19 were included for comparison, which were retrieved from a UMCG biobank including pre-donation samples of living kidney donors (IRB no. 2008/279). The absence of COVID-19 in healthy controls was evidenced by negative PCR testing for those samples retrieved during the pandemic (*n* = 2), whereas all other samples were retrieved before the COVID-19 pandemic (*n* = 28). The study was conducted in accordance with the principles of the Declaration of Helsinki (2013).

### 2.2. Data Collection

Demographic and clinical characteristics were collected for all participants, including age, gender, BMI, medical history, and smoking status. Medical history was categorized as hypertension, chronic cardiac disease (not hypertension), diabetes mellitus, and chronic pulmonary disease. In addition to serum free thiol measurements (vide infra), standard laboratory measurements were performed in all participants, including hemoglobin (Hb), C-reactive protein (CRP), white blood cell counts (WBC), platelet count, albumin, and creatinine. Hb, WBC count, and platelet count were measured using an automated hematology analyzer (Sysmex XE-2100, Sysmex Corporation, Kobe, Japan). Albumin and CRP were measured with turbidimetry using an automated analyzer (Roche Modular, Roche Diagnostics, Mannheim, Germany), whereas a photometric method was used to measure serum creatinine (Roche Modular, Roche Diagnostics, Mannheim, Germany). Furthermore, the estimated glomerular filtration rate (eGFR) was calculated using the Chronic Kidney Disease Epidemiology Collaboration (CKD-EPI) formula. In the non-hospitalized COVID-19 cohort, all laboratory measurements were performed at different time points, with the first time point (day 0) being within 48 h of positive PCR and the second time point (day 7) seven days after the initial time point. As for hospitalized COVID-19 subjects, only samples from day 0 were available and included in the analyses.

### 2.3. Measurement of Free Thiols

Serum free thiol concentrations were measured as previously described, though with minor modifications [18,19]. Serum samples were stored at −80 °C until further analysis to prevent significant changes in free thiol stability. First, samples were four-fold diluted using 0.1 M Tris buffer (pH 8.2). Background absorption was measured at 412 nm using the CLARIOstar Plus microplate reader (BMG Labtech, Ortenberg, Germany) concurrent with a reference measurement at 630 nm. Subsequently, 20 μL 1.9 mM 5,5′-dithio-bis(2-nitrobenzoic acid) (DTNB, Ellman’s reagent, CAS no. 69-78-3, Sigma-Aldrich Corporation, St. Louis, MO, USA) was added to the samples within 0.1 M phosphate buffer (pH 7.0). Samples were incubated for 20 min at room temperature, followed by a second measurement of absorption. Final concentrations of free thiols were determined by parallel measurement of an L-cysteine (CAS no. 52-90-4, Fluka Biochemika, Buchs, Switzerland) calibration curve (15.6–1000 μM) in 0.1 M Tris/10 mM EDTA (pH 8.2). Sample measurements demonstrated intra- and interday coefficients of variation (CV) <10%. Free thiol measurements in seven additional samples from subjects that were eventually hospitalized, were performed on another day and normalized to the data from the first day using internal experimental controls (correction factor (CF) = 0.82). Finally, serum concentrations of free thiols were adjusted for albumin (by calculating the free thiol groups/albumin ratio) as albumin is the most dominant plasma protein and therefore largely determines the quantity of potentially detectable free thiols [13].

### 2.4. Statistical Analysis

Baseline demographic, clinical, and biochemical characteristics were presented as means ± standard deviation (SD), medians [interquartile range (IQR)] in case of non-normal distributions, or proportions *n* with corresponding percentages (%). Assessment of normality was performed using histograms, normal probability plots (Q-Q plots), and Shapiro–Wilk normality tests. Differences between groups were assessed using independent sample *t*-tests or Mann–Whitney *U*-tests in case of continuous variables, whereas nominal variables were compared using chi-square tests or Fisher’s exact tests, as appropriate. Within-group comparisons for continuous normally distributed variables were performed using paired *t*-tests. Univariable and multivariable linear regression analyses were used to identify parameters that were associated with serum free thiol concentrations. Multivariable analysis was performed using backward elimination (*P*_OUT_ > 0.05) and included all statistically significantly associated variables from univariable analysis. Standardized beta (St. β) coefficients with corresponding *P*-values were reported, indicating magnitude, direction, and statistical significance of the observed associations. St. β coefficients represented the difference in serum free thiol concentrations per 1-SD increment or decrement for continuous variables or compared to the implied reference group for categorical variables. Skewed variables were log-transformed before entry into linear regression analyses. To evaluate the discriminative capacity of serum free thiol concentrations, classification analyses were performed by plotting receiver operating characteristics (ROC) curves while calculating the area under the curve (AUC) as an overall measure of classification performance. ROC statistics and AUCs were computed using the non-parametric, tie-corrected trapezoidal approximation method. Two-tailed *p*-values ≤ 0.05 were considered statistically significant. All data were analyzed using SPSS Statistics 23.0 software package (SPSS Inc., Chicago, IL, USA) and visualized using the Python programming language (v.3.7.12, Python Software Foundation), using the *pandas* (v.1.3.3), *matplotlib* (v.3.4.3), and *seaborn* (v.0.11.2) packages.

## 3. Results

### 3.1. Cohort Characteristics

Baseline demographic and clinical characteristics of non-hospitalized, mild COVID-19 subjects (*n* = 29) and healthy controls (HCs) (*n* = 30) are shown in Table 1. No significant differences in age (mean (SD) 59.8 ± 8.2 vs. 59.1 ± 8.0, *p* = 0.719), gender (58.6% vs. 60% females, *p* = 0.914), or body mass index (BMI) (26.5 ± 3.7 vs. 26.3 ± 3.8, *p* = 0.828) were observed between groups, indicating successful case-control matching. Furthermore, no significant differences were observed in smoking history (50% vs. 50% ever-smoker, *p* = 1.000) or comorbidities, including hypertension (13.8% vs. 20%, *p* = 0.731), chronic cardiac disease not including hypertension (6.9% vs. 10%, *p* = 1.000), diabetes mellitus (6.9% vs. 6.7%, *p* = 1.000), and chronic pulmonary disease (13.8% vs. 13.3%, *p* = 1.000). Multiple biochemical parameters showed significant differences between mild COVID-19 patients and HC, including increased concentrations of CRP (median (IQR) 1.9 (1.1–5.5) vs. 1.1 (0.7–1.4) mg/L, *p* = 0.041) and decreased concentrations of WBC (4.3 (3.6–5.9) vs. 5.6 (4.8–7.1) × 10^9^/L, *p* = 0.001) and albumin (44.0 ± 3.2 vs. 45.7 ± 2.2 g/L, *p* = 0.020) in patients with mild COVID-19. Among all COVID-19 subjects (*n* = 36, non-hospitalized and hospitalized), a diverse clinical symptomatology was observed, varying from asymptomatic (*n* = 2), mild (*n* = 23), moderate (*n* = 4), to severe symptoms (*n* = 7; hospitalized subjects). Six subjects (16.7%) developed long-term symptoms (i.e., long-COVID), among which five were non-hospitalized. Among all non-hospitalized COVID-19 subjects, the majority (*n* = 21) did not receive any treatment for COVID-19, whereas some subjects received supportive treatment with paracetamol (*n* = 3) or albuterol inhalation (*n* = 1). As for the hospitalized group, data on treatment prior to sample retrieval were available for only one subject, showing no COVID-19-related treatment use. For the rest of COVID-19 subjects, no information on COVID-19-related treatment was available.

### 3.2. Serum Free Thiol Concentrations Are Significantly Reduced in Patients with COVID-19

**Figure 1:**
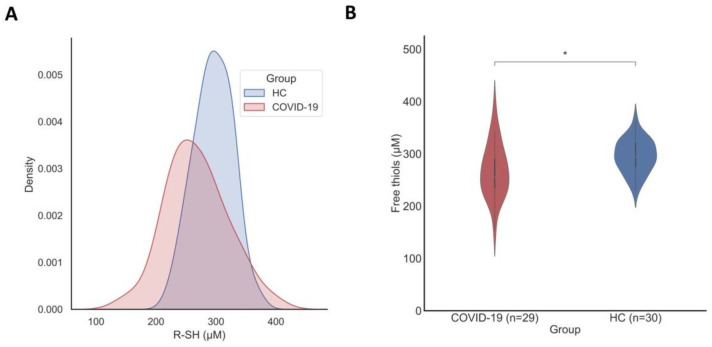
(**A**) Kernel density estimation of the distributions of serum free thiol concentrations among subjects with mild COVID-19 and healthy controls, demonstrating a normal distribution in both groups. Density estimates were performed using a Gaussian kernel. (**B**) Baseline serum concentrations of free thiols (µM) are significantly reduced in subjects with mild COVID-19 as compared to healthy controls (* *p* = 0.014).

Serum free thiol concentrations were significantly reduced in patients with mild COVID-19 (267.8 ± 50.4 μM, *p* = 0.014) compared to matched HCs (295.3 ± 31.2 μM) (Figure 1). Albumin-adjusted serum concentrations of free thiols showed a similar trend with lower concentrations in COVID-19 subjects (6.1 ± 0.9 μM/g) compared to HCs (6.4 ± 0.6 μM/g), although the difference did not reach statistical significance (*p* = 0.076) with these sample sizes. Serum concentrations of free thiols were normally distributed in both mild COVID-19 (*p* = 0.826) and healthy controls (*p* = 0.946), as assessed by visual inspection and confirmed by Shapiro–Wilk tests.

### 3.3. Associations of Serum Free Thiol Concentrations with Disease Parameters

**Figure 2:**
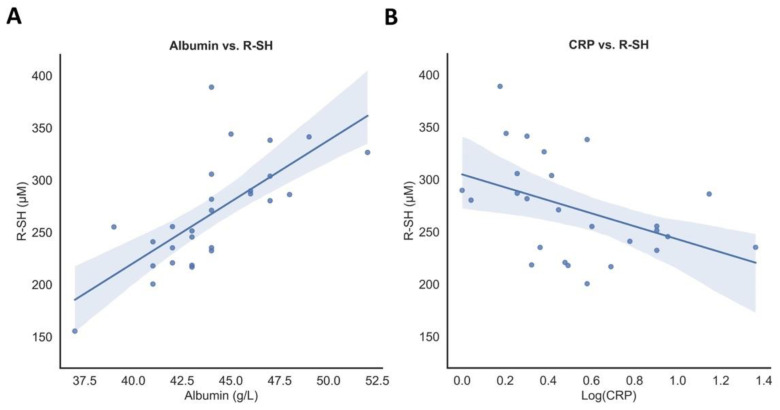
(**A**) Serum CRP concentrations are inversely associated with serum free thiol concentrations (R-SH) (St. β = −0.434, *p* = 0.027) in mild COVID-19 subjects. (**B**) Serum albumin is positively associated with serum free thiol concentrations (R-SH) (μM) (St. β = 0.710, *p <* 0.001) in mild COVID-19 subjects.

To identify parameters that were associated with serum free thiol concentrations, univariable and multivariable linear regression analyses were performed (Table 2). Univariable linear regression showed that both CRP (standardized beta (St. β) = −0.434, *p* = 0.027) and albumin concentrations (St. β = 0.710, *p <* 0.001) were significantly associated with serum free thiols in patients with mild COVID-19 (Figure 2). In multivariable linear regression analysis, albumin concentrations were independently associated with serum free thiol concentrations (St. β = 0.641, *p <* 0.001). CRP was not statistically significantly associated with albumin (St. β = −0.232, *p* = 0.254). Among HCs, age (St. β = −0.497, *p* = 0.005), eGFR (St. β = 0.481, *p* = 0.008), and albumin concentrations (St. β = 0.571, *p* = 0.001) showed an association with serum free thiol concentrations in univariable linear regression analyses, where age (St. β = −0.401, *p* = 0.011) and albumin concentrations (St. β = 0.494, *p* = 0.002) appeared to be independently associated in multivariable linear regression analysis.

### 3.4. Serum Free Thiol Concentrations Associate with COVID-19 Disease Severity

To examine the potential correlation between free thiols and COVID-19 disease severity, analyses were performed on serum free thiol measurements at different time points (day 0 vs. day 7) and in clinical subgroups, consisting of hospitalized (*n* = 7) and non-hospitalized patients (*n* = 29) (Table 3 and Table 4). Non-hospitalized patients with COVID-19 demonstrated statistically significantly lower concentrations of serum free thiols at day 7 (247.0 ± 48.5) compared to day 0 (267.8 ± 50.4, *p* = 0.001). Other laboratory parameters also showed significant differences between time point day 7 and time point day 0, including decreased concentrations of hemoglobin (8.9 ± 0.7 g/dL vs. 9.1 ± 0.7, *p* = 0.006), albumin (42.6 ± 3.0 vs. 44.0 ± 3.2 g/L, *p* = 0.001), and creatinine (69.0 (59.0–82.0) vs. 71.0 (61.0–80.0) μmol/L, *p* = 0.005), as well as increased eGFR (88.9 ± 11.9 vs. 85.1 ± 13.4 mL/min × 1.73 m^2^, *p* = 0.010).

No statistically significant difference in baseline serum free thiol concentrations was found between subjects who eventually required hospitalization (262.1 [206.4–268.1]) and those non-hospitalized (255.5 [233.8–296.8], *p* = 0.325). Hospitalized COVID-19 patients demonstrated statistically significantly higher concentrations of CRP (48 [9.0–81.0] vs. 1.9 [1.0–5.5], *p <* 0.001) and creatinine (91.8 [85.5–97.2] vs. 71.0 [61.0–80.0], *p* = 0.022) and lower albumin (39.1 [37.7–42.1] vs. 44.0 [42.0–46.0], *p* = 0.010).

### 3.5. Discriminative Capacity of Serum Free Thiols as Biomarker for COVID-19 Disease Severity

**Figure 3:**
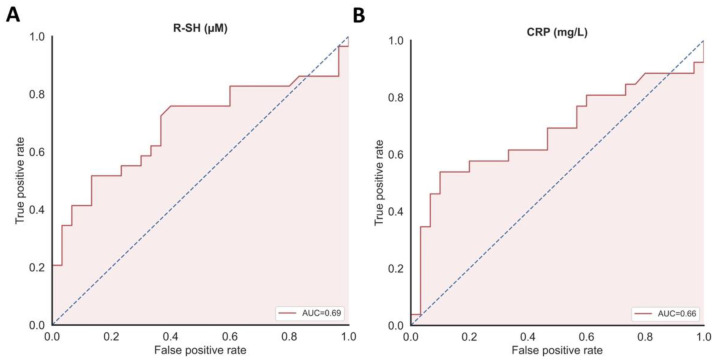
(**A**) Serum concentrations of free thiols (µM) significantly discriminated between patients with COVID-19 and healthy controls and (**B**) showing a slightly higher discriminative capacity as compared to CRP concentrations (mg/L).

Serum free thiol concentrations were evaluated for their discriminative ability to differentiate between study groups (non-hospitalized COVID-19 vs. HC) (Figure 3). Serum concentrations of free thiols significantly discriminated between patients with mild COVID-19 and healthy controls [area under the curve (AUC) = 0.69, 95% confidence interval (CI) = 0.55–0.83, *p* = 0.011). The discriminative capacity of CRP concentrations regarding COVID-19 was slightly lower than that of serum free thiol concentrations (AUC = 0.66, CI 0.51–0.81, *p* = 0.042) or CRP and serum free thiol concentrations combined (AUC = 0.68, CI 0.53–0.83, *p* = 0.020). Since albumin was shown to be a strong determinant of free thiol concentrations, the discriminative capacity of albumin-adjusted free thiols was also determined and appeared to be slightly lower than free thiols alone (AUC = 0.67, CI 0.52–0.81, *p* = 0.032). The discriminative performance of baseline concentrations of serum free thiols with regard to hospitalized and non-hospitalized COVID-19 subjects, however, was rather low and not statistically significant (AUC = 0.63, 95% CI 0.42–0.83, *p* = 0.308), most likely due to the small sample size of hospitalized individuals (*n* = 7).

## 4. Discussion

In this study, we examined the association between serum free thiol concentrations—as a biomarker for systemic oxidative stress—and COVID-19 in a cohort primarily consisting of non-hospitalized individuals. Most notably, we demonstrated that serum free thiols were significantly lower in COVID-19 subjects as compared to healthy controls, providing valuable evidence of the presence of a systemic redox imbalance in COVID-19 even in relatively milder cases not requiring hospital admission. This finding is a valuable addition to the previously observed presence of oxidative stress in severe COVID-19 cases by others (vide infra) [8]. Importantly, the difference in serum free thiols between COVID-19 subjects and healthy controls was clearly present even with successful matching on age, gender, and BMI, all of which have previously been shown to affect free thiol concentrations [10,20]. Our findings further improve the understanding of COVID-19 pathophysiology and fuel the rationale for antioxidant treatment modalities.

In this context, the association between redox status and severity of COVID-19 may also be relevant. To examine a potential association, serum free thiol concentrations were compared between two different time points (day 0 vs. day 7), as COVID-19 is a dynamic disease with usually initial progression of illness after symptom onset. Free thiols were indeed significantly lower at day 7 compared to day 0, suggesting the involvement of oxidative stress in the early progression of disease. As would be expected in response to fighting off the SARS-CoV-2 infection, a decline in hemoglobin and albumin concentrations was observed, indicating disease progression with more severe disease at day 7 compared to day 0. Accordingly, CRP appeared to slightly increase, although this was not statistically significant. Kidney function, however, appeared to improve over time, which was a rather surprising finding. No statistically significant association was observed between serum free thiol concentrations and hospitalization. However, sample size was small in the hospitalized group (*n* = 7), reasonably resulting in insufficient power to demonstrate such an association.

A previous study among 144 COVID-19 patients investigated thiol-disulphide homeostasis as an indicator of oxidative stress in this disease [8], using a spectrophotometric method developed by Erel and Neselioglu to measure native thiol, total thiol, and disulphide levels [16]. Similar to our results, this study demonstrated lower free thiol concentrations in COVID-19 patients (242.1 ± 90.0, *p <* 0.001) as compared to healthy controls (419.8 ± 55.9) and free thiols appeared to be a useful marker for COVID-19 diagnosis, but the authors additionally found this decrease in thiol concentrations to be predictive of disease severity. Of note, the study did not take BMI into account in the performed analyses, while this may be particularly relevant as BMI has been shown to strongly correlate with free thiols in other conditions [9,10,11] and COVID-19 patients who are overweight or obese are more prone to develop severe disease [21]. Therefore, control matching in the current study included BMI in addition to age and gender.

In the current study, we found an independent association between serum free thiol concentrations and albumin concentrations in both COVID-19 subjects and healthy controls. The positive correlation between serum free thiols and albumin concentrations can be explained by the fact that extracellular free thiols mainly consist of circulating cysteine-based proteins, of which albumin is the most relevant example, as it is quantitatively the most relevant plasma protein, but also because of its redox properties (the single free Cys^34^ thiol residue) [13,22,23]. For these reasons, previous studies investigating free thiols in a variety of disease contexts have accounted for systemic albumin concentrations as this largely determines the level of potentially detectable free thiols [9,10,24]. In the current study, albumin-adjusted serum free thiol concentrations appeared to be lower in COVID-19 subjects as compared to HCs. However, although close-to-significant, the observed difference did not reach statistical significance with these sample sizes. Considering this, the observed findings of lowered serum free thiol concentrations among subjects with COVID-19 may (at least partially) be explained by their significantly lower albumin concentrations. Hypoalbuminemia is commonly observed in COVID-19 and has been associated with disease severity, possibly induced by systemic inflammation with increased capillary permeability [25]. The interrelation between hypoalbuminemia and increased levels of inflammation and oxidative stress has been recognized before and is likely to be involved in the progression of COVID-19 [26]. The relationship of inflammation and oxidative stress is further supported by our finding of a negative association between CRP and serum free thiol concentrations in COVID-19 patients, an association which has been observed in other conditions too [10,27], which can be explained by ROS production by inflammatory cells and oxidative stress-induced activation of transcription of pro-inflammatory mediators. A similar negative association between CRP and albumin would be expected in case lower albumin concentrations were the sole reason of the observed lower serum free thiol concentrations, but this association appeared to be less strong and not statistically significant, pointing out that the observed difference in serum concentrations of free thiols are not entirely accounted for by albumin.

As free thiols are a reliable marker for oxidative stress, their quantification in COVID-19 may form a promising and non-invasive strategy to identify subjects with impaired redox status in order to rationally select which individuals would benefit the most from antioxidant treatment [5,28,29]. On this account, hydrogen sulfide (H_2_S) or its precursor N-acetylcysteine (NAC)—among other antioxidant treatment modalities—have been implicated as potentially beneficial supplementary treatments in COVID-19 by controlling redox homeostasis, in addition to their anti-inflammatory and antiviral effects [6]. For example, reduced serum H_2_S levels have been associated with higher mortality in patients hospitalized because of COVID-19 pneumonia, while administration of NAC was recently shown to reduce the risk for mechanical ventilation and mortality in a retrospective, two-center cohort study [30,31]. Similarly, taurine has been considered a potential disease-modulating treatment in COVID-19, based on its capacity to augment endogenous H_2_S production, as well as its own antioxidant and anti-inflammatory activity [6,32]. Antioxidant therapy has been proven to be beneficial in conditions associated with redox imbalance (e.g., sepsis, ARDS, acute lung injury), demonstrating its potential as putative therapy in COVID-19. Treatment with resveratrol may be beneficial in this regard, considering its antioxidant properties with literature showing increased thiol levels in rats and in patients [33,34,35], in addition to its direct antiviral effects [36]. Other promising results in COVID-19 were demonstrated with the use of quercetin, a flavonoid derived from plants, as well as N-acetyl-5-methoxytryptamine (also known as melatonin), a tryptophan derivative produced in the pineal gland and immune cells, which both are known to act as a free radical scavenger, in addition to their immunomodulatory and anti-inflammatory actions [37,38]. Finally, several vitamins and minerals have been reported as promising supplementary treatment options in COVID-19 due to their antioxidant properties, including vitamin C, vitamin E, and zinc, among others [39,40]. However, thiol-targeted antioxidants should ideally not unfavorably interfere with the physiological functions of ROS, but rather stay reserved for individuals who demonstrate clear overproduction of ROS [41]. The latter phenomenon may be reflected by serum free thiols, serving as a minimally invasive marker of systemic oxidative stress.

This study has several strengths and limitations that should be addressed. Most importantly, this study provides convincing evidence of a significant association between serum free thiol concentrations—being a minimally invasive method to quantify systemic oxidative stress—and COVID-19, as these concentrations were evidently reduced in non-hospitalized subjects with relatively mild COVID-19. This cohort of non-hospitalized subjects is unique and provides new evidence on the presence of oxidative stress in COVID-19, complementing previous studies [7,8], but this time specifically for milder cases. The short-term follow-up with clinical and biochemical data available at both day 0 and day 7 allowed us to make observations over time in the disease course. At the same time, however, the small sample size possibly prevented the demonstration of potential associations (e.g., between free thiols and disease severity) with smaller effect sizes. Furthermore, it would have been more optimal if samples from the COVID-19 subjects were collected at the exact same time point as the control patients. A larger confirmatory study is needed to validate our results. Nevertheless, we think that our preliminary results may help guide future performance of a larger-scale study.

## 5. Conclusions

In conclusion, we demonstrate that non-hospitalized individuals with COVID-19 are markedly affected by systemic oxidative stress, as reflected by reduced serum free thiol concentrations in comparison with age-, sex-, and BMI-matched healthy controls. Furthermore, we show that serum free thiols strongly associate with circulating albumin and CRP, the latter being fairly comparable to serum free thiols in terms of discriminative capacity. Future studies are warranted to externally validate serum free thiols in larger, prospective patient cohorts of COVID-19, while simultaneously assessing their value in relation to disease course and as non-invasive read-out for oxidative stress in this disease.

## Figures and Tables

**Table 1:** Baseline demographic and clinical characteristics of subjects with mild COVID-19 and matched healthy controls.

	COVID-19		HC	*p*-Value
	*n* = 29		*n* = 30	
Age (years)	59.8 ± 8.2		59.1 ± 8.0	0.719
Female, *n* (%)	17 (58.6)		18 (60)	0.914
BMI (kg/m^2^)	26.5 ± 3.7		26.3 ± 3.8	0.828
Ever smoker, *n* (%)	14 (50) *		15 (50)	1.000
Comorbidities				
Hypertension, *n* (%)	4 (13.8)		6 (20)	0.731
Chronic cardiac disease (not hypertension), *n* (%)	2 (6.9)		3 (10)	1.000
Diabetes mellitus, *n* (%)	2 (6.9)		2 (6.7)	1.000
Chronic pulmonary disease, *n* (%)	4 (13.8)		4 (13.3)	1.000
Laboratory measurements				
Serum free thiols (μM)	**↓**	267.8 ± 50.4	295.3 ± 31.2	**0.014**
Hemoglobin (g/dL)		8.9 [8.7–9.4]	8.9 [8.4–9.4]	0.601
CRP (mg/L)	**↑**	1.9 [1.1–5.5]	1.1 [0.7–1.4]	**0.041**
WBC (×10⁹/L)	**↓**	4.3 [3.6–5.9]	5.6 [4.8–7.1]	**0.001**
Platelets (×10⁹/L)		228.1 ± 60.8	252.6 ± 68.5	0.157
Albumin (g/L)	**↓**	44.0 ± 3.2	45.7 ± 2.2	**0.020**
eGFR (mL/min × 1.73 m²)		86.0 [78.0–95.0]	86.0 [74.5–96.0]	0.967
Creatinine (μmol/L)		71.0 [61.0–80.0]	75.0 [65.5–86.0]	0.277

Data are presented as means ± SD, median [IQR] or proportions *n* with corresponding percentages (%). * Case with missing values (*n* = 1) was excluded from analysis. Red arrows indicate significantly reduced concentrations in COVID-19 subjects; green arrows indicate significantly elevated concentrations in COVID-19 subjects. Abbreviations: BMI, body mass index; COVID-19, coronavirus disease 2019; CRP, C-reactive protein; eGFR, estimated glomerular filtration rate; HC, healthy controls; WBC, white blood cell count.

**Table 2:** Univariable and multivariable linear regression analyses of serum free thiol concentrations with clinical and biochemical parameters in COVID-19 subjects and healthy controls.

	COVID-19				HC			
	Univariate Analysis		Multivariate Analysis		Univariate Analysis		Multivariate Analysis	
	St. β *	*p*-Value	St. β *	*p*-Value	St. β *	*p*-Value	St. β *	*p*-Value
Age	−0.178	0.357			−0.497	**0.005**	−0.401	0.011
Gender	−0.019	0.920			−0.220	0.242		
BMI	−0.258	0.185			−0.027	0.887		
Laboratory measurements								
Hemoglobin (g/dL)	0.183	0.353			0.345	0.062		
CRP (mg/L)	−0.434	**0.027**			−0.280	0.134		
WBC (×10⁹/L)	0.215	0.272			−0.247	0.188		
Platelets (×10⁹/L)	0.129	0.514			0.029	0.880		
Albumin (g/L)	0.710	**<0.001**	0.641	**<0.001**	0.571	**0.001**	0.494	**0.002**
eGFR (mL/min × 1.73 m²)	0.147	0.463			0.481	**0.008**		
Creatinine (μmol/L)	−0.017	0.932			−0.201	0.288		

* Standardized beta (St. β) coefficient representing the difference in serum free thiol concentrations per 1-SD increment/decrement for continuous variables and the difference in serum free thiol concentrations compared to the implied reference group for categorical variables. Abbreviations: BMI, body mass index; COVID-19, coronavirus disease 2019; CRP, C-reactive protein; eGFR, estimated glomerular filtration rate; HC, healthy controls; WBC, white blood cell count. **Bold** *p*-values indicate statistical significance (<0.05).

**Table 3:** Serum free thiol concentrations and other laboratory measurements in mild COVID-19 subjects at different time points.

	COVID-19, Day 0	COVID-19, Day 7		*p*-Value
	*n* = 29	*n* = 29		
Serum free thiols (μM)	267.8 ± 50.4	**↓**	247.0 ± 48.5	**0.001**
Hemoglobin (g/dL)	9.1 ± 0.7	**↓**	8.9 ± 0.7	**0.006**
CRP (mg/L)	1.9 [1.0–5.5]		2.9 [0.9–9.5]	0.170
WBC (×10⁹/L)	4.3 [3.6–5.9]		4.8 [4.0–6.3]	0.063
Platelets (×10⁹/L)	228.1 ± 60.8		249.3 ± 57.7	0.117
Albumin (g/L)	44.0 ± 3.2	**↓**	42.6 ± 3.0	**0.001**
eGFR (mL/min × 1.73 m²)	85.1 ± 13.4	**↑**	88.9 ± 11.9	**0.010**
Creatinine (μmol/L)	71.0 [61.0–80.0]	**↓**	69.0 [59.0–82.0]	**0.005**

Data are presented as means ± SD, median [IQR] or proportions *n* with corresponding percentages (%). Red arrows indicate significantly reduced concentrations at day 7; green arrows indicate significantly elevated concentrations at day 7. Abbreviations: COVID-19, coronavirus disease 2019; CRP, C-reactive protein; eGFR, estimated glomerular filtration rate; HC, healthy controls; WBC, white blood cell count. **Bold** *p*-values indicate statistical significance (<0.05).

**Table 4:** Serum free thiol concentrations and other laboratory measurements in non-hospitalized COVID-19 subjects and COVID-19 subject who eventually required hospitalization.

	Non-Hospitalized	Hospitalized		*p*-Value
	*n* = 29	*n* = 7		
Serum free thiols (μM)	255.5 [233.8–296.8]		262.1 [206.4–268.1]	0.325
Hemoglobin (g/dL)	8.9 [8.7–9.4]		8.8 [7.9–9.4]	0.479
CRP (mg/L)	1.9 [1.0–5.5]	**↑**	48.0 [9.0–81.0]	**<0.001**
WBC (×10⁹/L)	4.3 [3.6–5.9]		4.8 [3.4–7.7]	0.952
Platelets (×10⁹/L)	230.0 [169.3–265.8]		173.0 [144.0–207.0]	0.079
Albumin (g/L)	44.0 [42.0–46.0]	**↓**	39.1 [37.7–42.1]	**0.010**
eGFR (mL/min × 1.73 m²)	86.0 [78.0–95.0]		69.1 [68.9–84.1]	0.109
**Creatinine (μmol/L)**	71.0 [61.0–80.0]	**↑**	91.8 [85.5–97.2]	**0.022**

Data are presented as means ± SD, median [IQR] or proportions *n* with corresponding percentages (%). Red arrows indicate significantly reduced concentrations in hospitalized COVID-19 subjects; green arrows indicate significantly elevated concentrations in hospitalized COVID-19 subjects. Abbreviations: COVID-19, coronavirus disease 2019; CRP, C-reactive protein; eGFR, estimated glomerular filtration rate; HC, healthy controls; WBC, white blood cell count. **Bold** *p*-values indicate statistical significance (<0.05).

## Data Availability

All data is contained within the present article.
